# The association between preoperative epidural steroid injections and postoperative cervical and lumbar surgical site infections: A systematic review and meta-analysis

**DOI:** 10.1016/j.xnsj.2024.100334

**Published:** 2024-06-05

**Authors:** David Sherwood, Jakob Dovgan, Derek Schirmer, R. Sterling Haring, Byron Schneider

**Affiliations:** a University Health Lakewood Medical Center, Department of Orthopedics; Kansas City, MO, United States; b Vanderbilt University Medical Center, Physical Medicine and Rehabilitation Department; Nashville, TN, United States; c Stanford University Medical Center, Physical Medicine and Rehabilitation Division, Redwood City, CA, United States; d Department of Pain Medicine, Southwell Medical, Tifton, GA, United States; e Department of Health Policy and Management, Johns Hopkins Bloomberg School of Public Health, Baltimore, MD, United States

**Keywords:** Spine, Infection, Epidural, Steroid, Spinal stenosis, Surgery, Injection

## Abstract

**Background:**

Is there a statistically significant association between preoperative epidural steroid injections (ESI) and postoperative cervical and lumbar spinal surgery infections (SSI)?

**Methods:**

A systematic review and meta-analysis was completed of patients 18 years or older who underwent elective cervical or lumbar spinal surgery. Those who underwent surgery with preoperative ESI were compared to those without. We assessed for differences in postoperative SSI incidence. Electronic literature databases were searched through October 2022. Peer-reviewed publications that included raw data regarding epidural exposure and non-exposure were included. Case reports, case series, abstracts, editorials, or publications that did not include raw data were excluded. Odd's ratios (OR) were calculated from the raw data collected. Meta-analysis was done using RevMan v5 with a fixed effects model.

**Results:**

We identified 16 articles for inclusion. When not controlling for the type of surgery and time from ESI to surgery, there was a statistically significant OR between preoperative ESI and postoperative SSI. The association persisted when the ESI was performed within 30 days or 31-90 days of the surgery. No association was discovered when evaluating only cervical spine surgeries. The evidence is assigned a “moderate” GRADE rating.

**Conclusions:**

Our analysis shows a small, time-dependent, statistically significant association between preoperative ESI and postoperative lumbar SSI may exist. However, the OR produced, while statistically significant, are close enough to 1.0 that clinically, the effect size is “small.” The number needed to treat for an ESI in the appropriate clinical setting is, at worst, 3. The number needed to harm, meaning the number of patients who undergo an ESI at any time before their spine surgery and then develop a SSI, is 111 patients. Ultimately, the surgical sparing potential from an ESI outweighs the SSI risk based on our findings.

## Introduction

There is inherently an increased risk of infection with any injection. The number of published articles on preoperative epidural steroid injections and postoperative surgical site infections is sparse. An insurance database review showed that 46.4% of patients had a lumbar epidural steroid injection within a year before lumbar spinal surgery due to a disc herniation or spinal stenosis [1]. Another meta-analysis of patients undergoing cervical, thoracic, or lumbar spine surgery, regardless of ESI usage, for any indication produced a pooled incidence for surgical site infection at 3.1% (95% CI 2.3 – 4.3%) [2]. The study did not stratify risk based on preoperative epidural steroid injection. There has been a reported 1.93 greater direct cost to treat patients with a surgical site infection when compared to those that do not [3]. In dollars, the cost to the patient may equate to an increase from $15,817 to $38,701 [4]. Thus, identifying and quantifying such a modifiable risk factor for postoperative infection could offer improved postoperative courses and reduced cost of care.

Unlike peripheral joint injections and arthroplasty, there are no guidelines to help patients and clinicians make decisions regarding preoperative epidural steroid injections and the risk of postoperative spine surgery infection. There have been 2 meta-analyses published that suggest restriction of preoperative epidural steroid injections to reduce postoperative spinal surgery infection [5,6]. Importantly, each meta-analysis used different studies, drew different conclusions, and produced differing recommendations. Kazarian 2021 recommended against all corticosteroid injections within 1 month of any spinal surgery. Patel 2022 recommended against only epidural steroid injections within 1 month of lumbar fusion surgery.

After reviewing both studies, these authors felt an additional analysis was warranted, given the conclusions drawn, statistical decisions, and study inclusion/exclusion.

## Objective

We sought to review the literature via a systematic review followed by a meta-analysis of multiple sub-cohorts so that clinicians would have data available to inform decision-making at the most granular level possible based on the published data.

## Methodology

### Population

Adults aged 18 years or older who underwent elective cervical or lumbar spine surgery (discectomy, decompression, and/or fusion).

### Intervention

Epidural steroid injections given at any time before their operation.

### Comparison

No epidural steroid injection was given at any time before their operation.

### Outcome

Surgical site infection.

### Studies

This review was restricted to randomized controlled trials, observational cohort studies, database reviews, meta-analyses, and systematic reviews, which included raw data about their exposure and nonexposure groups. Case reports, case series, abstracts, editorials, or studies that did not include raw data were excluded.

## Registration, sources, and search

This IRB-exempt study was registered on PROSPERO (International Prospective Register of Systematic Reviews, CRD42022375157) on November 23, 2022. Clinical outcome studies on the association between preoperative epidural steroid injections and postoperative spinal surgery site infections were obtained by searching PubMed, Cochrane Database of Systematic Reviews, and SCOPUS. The primary author designed the search strategy in consultation with a medical research librarian specializing in Systematic Review formulation at the University of Missouri-Columbia to search publications before October 1, 2022. Search results were uploaded to Zotero for screening and data extraction. The search strategies and date of search are presented in Appendix 1.

## Study selection

**Figure. 1:**
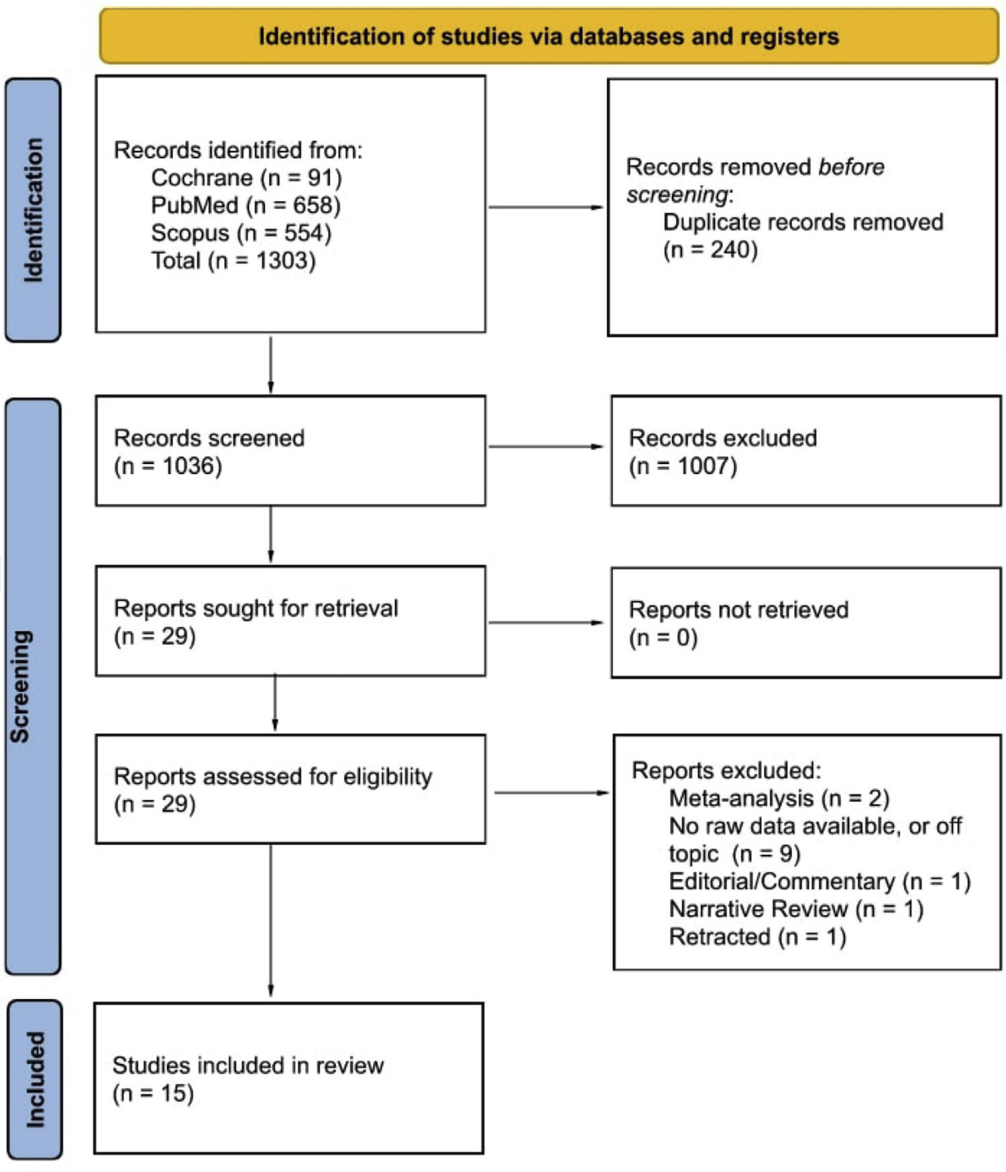
ESI SSI Prisma diagram.

Two authors with formal training and certification in the principles of evidence-based medicine independently assessed the titles and abstracts for relevance and eligibility. The primary author resolved discrepancies. Subsequently, 3 authors independently reviewed the selected publications in full and evaluated them for inclusion. The primary author resolved discrepancies. Additionally, the references were reviewed to assess whether further studies should be included. Studies were included if they presented clinically relevant data on the relationship between preoperative epidural steroid injections and postoperative spinal surgical site infections. Moreover, the studies were excluded if they did not provide raw data which could be used for statistical analyses (Fig. 1).

## Data items and collection

Reviewers extracted the following data from each study: (1) bibliographic details, (2) study design, (3) the source of the data (for example, single site, multi-site, database), (4) the surgery performed, (5) sample size (6) raw data between the 2 groups of those who did receive an epidural steroid presurgical intervention and those who did not, (7) published Odds Ratio and statistical significance, and (8) any reported timing between when the epidural was given preoperatively. Additionally, commentary was kept regarding aberrancies in methodology or statistics, selected Current Procedural Terminology (CPT)/International Classification of Diseases (ICD) codes, patient populations, and outcomes that may suggest bias.

## Data extraction

Articles that presented raw data regarding the number of patients with preoperative epidural steroid injections who had postoperative surgical site infections, patients with preoperative epidural steroid injections who did not have postoperative surgical site infections, patients without preoperative epidural steroid injections who had postoperative surgical site infections, and patients without preoperative epidural steroid injections who did not have postoperative surgical site infections had their data transferred into a password protected digital spreadsheet.

Articles that presented raw but incomplete data were evaluated to see if complete data could be curated by creating a 2×2 contingency table based on the published raw data in conjunction with published odds ratios, such that the missing data could be calculated by a reverse odds ratio calculation.

In scenarios in which time-matched exposure groups (0-30 days, 31-90 days, etc.) were compared against a single control group that was not time-matched for each respective cohort, the inclusion of such comparison data was deemed inappropriate for a subcohort time-based meta-analyses but appropriate for non-time matched meta-analyses.

Three authors independently reviewed each study for data. Discrepancies between authors were rectified by a collaborative meeting in which the data in question was reviewed between the parties to determine why the discrepancy occurred and then fix the discrepancy.

## Summary measures and synthesis of results

The primary outcome of interest was the odds ratio association with preoperative epidural steroid injections and postoperative spinal surgical site infection. For statistical purposes, studies were to be grouped by ESI, timing from injection to surgery, location of surgery, type of surgery, and the etiology of the data acquired (i.e., database, cohort).

## Risk of bias and methodological assessment

The body of evidence was evaluated using the Grades of Recommendation, Assessment, Development, and Evaluation (GRADE) appraisal system to determine the evidence's quality regarding the association. The GRADE system transparently evaluates the body of evidence in domains including risk of bias, imprecision, inconsistency, indirectness, and publication bias. GRADE provides an initial rating of quality based on the best available evidence. It allows for upgrading (*e.g.,* large magnitude of effect, dose-response gradient) or downgrading (*e.g.,* risk of bias, indirectness) of the evidence quality. Two authors independently reviewed the selected literature and provided assessments. A collaborative discussion between the 2 authors resolved disagreements regarding GRADE evaluation.

## Statistical methodology

RevMan v5.4.1 was used for data synthesis and meta-analysis. Incidence and population data were extracted from published manuscripts. Meta-analysis was performed using fixed effects, and alpha was set at 0.05.

## Results

### Systematic review

There were 1,303 articles initially identified. After removing duplicates (240), 1,036 articles were screened via abstract and title review. A total of 29 articles were deemed eligible for full review. 14 articles were excluded due to a lack of raw published data or lack of clarity on preoperative epidural steroid injections and postoperative spinal surgical site infections (9), retracted (1), editorial/commentary (1), meta-analyses (2), and a narrative review (1). Thus, 15 articles were identified for inclusion in the Systematic Review. The PRISMA flow chart visualization of this process is Fig. 1 [1,[7], [8], [9], [10], [11], [12], [13], [14], [15], [16], [17], [18], [19]]. Two studies were included in the systematic review but excluded from the meta-analysis due to statistical, methodological, or publication issues that could not be reconciled [12,20].

### Retrospective cohort studies

Zusman et al.was a single-center retrospective study of patients who underwent elective thoracic and lumbar spine fusion surgery, not for trauma, tumor, or infectious reasons, from 2007 to 2010. Among other complications, they evaluated postoperative surgical site infections captured within 30 days of the index surgery. They define epidural steroid injection by patient report of preoperative “spinal injection” at any time point. They do not delineate if that injection was an epidural injection, facet injection, medial branch block, trigger point injection, radiofrequency ablation, intramuscular injection, or other injection. Despite this, all patient-reported spinal injections are referred to as epidural steroid injections in the manuscript's title, abstract, results, and discussion. They found a nonstatistically significant increase in risk associated with preoperative patient-defined spinal injections and postoperative surgical site infection within 30 days of the index lumbar fusion. They did add that the complications did not affect outcome measures [7].

Hartveldt et al. was a dual-center retrospective study of patients who underwent lumbar spine surgery, both fusion and non-fusion, for degenerative lumbar spine conditions from 2005 to 2015 at Massachusetts General Hospital. They evaluated preoperative epidural steroid injections at 0-30 days, 30-90 days, and 0-90 days from surgical intervention to assess for postoperative surgical site infections. The identified preoperative epidural steroid injections using CPT codes. Among the selected CPT codes, 0217T was included. CPT 0217T is for ultrasound-guided facet injections, not lumbar epidural steroid injections (See Table 3). They defined surgical site infections as requiring reoperation or incision and drainage due to infection within 90 days of the index surgery. They identified no statistically significant association between preoperative steroid injections at 0-30 days, 30-90 days, and 0-90 days and postoperative surgical site infection within 90 days of the index surgery [8].

Ozturk et al. was a single-site study out of Turkey that evaluated patients who had undergone unilateral, single-level lumbar microdiscectomies due to extruded or sequestered disc material. While many variables were captured, they evaluated whether preoperative epidural steroid injections, defined as a mix of 80 mg triamcinolone and 3 mL of .5% bupivacaine injected into the anterior epidural space via a transforaminal approach, altered the risk of postoperative surgical site infections. Of note, it is the only study that defines the injectate's specifics. While they did not explicitly define surgical site infection, no infections were reported in the steroid or control groups. Given a lack of events, this individual study could not calculate an odds ratio. Ultimately, they were unable to find a difference between preoperative transforaminal epidural steroid injections given at 0-30 days, 30-60 days, 60-180 days, 180-365 days, or >365 days and postoperative surgical site infections [13].

Kreitz et al. was a retrospective single-site study from Philadelphia, PA, which looked at the risk of preoperative ESI and postoperative SSI in patients who had undergone lumbar decompression or lumbar fusion performed for a diagnosis of lumbar radiculopathy and/or spinal stenosis with minimum 90 days follow up. They defined preoperative ESI via the following CPT codes: 62311, 64475, 64483, and 64493. However, 64475 and 64493 are CPT codes for facet injections (See Table 3). Therefore, the study's results included procedures that are not epidural steroid injections. They used ICD codes 996.67, 998.12, 998.31, 998.32, 998.59, T81.31XA, T81.32XA, and T84.7XXA to define surgical site infection. The study does not delineate the type of epidural performed, the type of steroids used, or the dosage of the injectate. There was no statistically significant difference between the ESI and control group for the lumbar decompression without fusion group. There was a statistically significant difference between the ESI and control group for the lumbar fusion group [15].

Shakya et al. was a retrospective single-site study from India that evaluated patients aged 21-65 who had undergone lumbar discectomy for single-level disc herniations from 2017 to 20. 129 patients received a transforaminal epidural steroid injection at 0-90 days, 90-180 days, and >180 days before moving onto surgery, while 186 patients went directly to lumbar discectomy. They do not define the injectate or dose for the transforaminal epidural steroid injection. They do not define SSI but report that identification of infection was determined by retrospective chart review. There was no statistically significant difference between the 2 groups concerning surgical site infection. Interestingly, all 129 patients who received a transforaminal epidural steroid injection preoperatively matriculated onto lumbar discectomy. Zero patients found “substantial relief” with preoperative transforaminal epidural steroid injection [17].

### Retrospective database studies

Cancienne et al. is a retrospective analysis of a Medicare PearlDiver Patient Record Database that was used to compare postoperative infection rates within 90 days for patients who had undergone an anterior cervical discectomy and fusion (ACDF) or posterior cervical fusion (PCF) and had a preoperative cervical epidural steroid injection from 2005 to 2012. CPT Codes 22554, 22551, 22585, 22590, and 22600 were used to identify patients with either an ACDF or PCF. CPT codes 64479 and 62310 were used to identify patients with a preoperative cervical epidural steroid injection. The exposure group was controlled for 0-90 days, 91-180 days, and 181-365 days. The study defines surgical site infection as a postoperative infection within 90 days and uses the CPT codes 20005, 10180, 21501 or ICD-9: 998.5, 998.51, 998.59, 996.67, 996.69 to search the database. There was a statistically significant association for post-ACDF SSI when preoperative cervical epidural steroid injections were given 0-90 days before the operation. There was a statistically significant association for post-PCF SSI when preoperative cervical epidural steroid injections were given 0-180 days before the operation [19].

Singla et al. was a retrospective study that utilized the Medicare PearlDiver Database from 2005 to 12 to evaluate preoperative ESI association with postoperative SSI related to 1-2 level posterior lumbar fusion surgery. Lumbar surgeries were identified via CPT codes. Lumbar epidural steroid injections were identified via CPT codes. Surgical site infections were defined by ICD-9 codes within 90 days of the index lumbar fusion surgery. They found a statistically significant association between postoperative lumbar fusion surgery surgical site infection and preoperative lumbar epidural steroid injections at 0-30 days and 31-90 days, but not 91-180 days before the surgery [11].

Yang et al. was a retrospective study that utilized the Medicare PearlDiver Database from 2005 to 12 to evaluate preoperative lumbar epidural steroid injections association with preoperative SSI as it relates to 1-2 level lumbar decompression surgery. Lumbar surgeries were identified via CPT codes 63005, 63030, 63047. Lumbar epidural steroid injections were identified via CPT codes 64483 and 62311. Patients who received facet interventions were excluded. Surgical site infections were defined by ICD-9 codes 998.5, 998.51, 998.59, and CPT codes 20005 and 22015 within 90 days of the index lumbar decompression surgery. Surgeries that preoperatively involved infection or tumor were excluded. They found a statistically significant risk for postoperative surgical site infection associated with preoperative lumbar epidural steroid injections at 0-30 days and 31-90 days before the operation but found no statistically significant association when the injection was given >90 days from the operation [9].

Donnally et al. produced a nearly identical study to Yang et al. Donnally and Yang defined the surgery using the 63030 and 63047 CPT codes, but Yang added the 63005 CPT code for both 1 and 2-level decompressions. Using the Medicare PearlDiver Database, Donnally reviewed from 2005 to 14, while Yang reviewed from 2005 to 12. They used identical codes for epidural steroid injections: 64483, 62311. They defined surgical site infections using ICD 9 codes 998.5, 998.51, 998.59, CPT Code 20005, and 22015. The only difference in the definition of a surgical site is that Donnally added 996.67, which is infection/inflammation from an orthopedic device, implant, or graft. Despite the similarities, Donnally 2018 found a statistically significant association between preoperative epidural steroid injections and postoperative surgical site infections for epidural steroid injections at 31-90 days and 91-180 days preoperative. Unlike Yang, Donnally found no statistically significant association with ESI given within 30 days of surgery. In summary, these 2 studies used similar search methods in the same database over a similar period. However, Yang found statistically significant risk at 0-30 days and 31-90 days, while Donnally found risk at 31-90 days and 91-180 days [9,12]. The only agreement was between the increased risk associated with a preoperative epidural steroid injection at 31-90 days.

Given the overlap between the Donnally and Yang study, including both studies for the meta-analysis is inappropriate, given that an assumed large percentage of the data will then be counted twice without a way to differentiate such data. Including the Yang data eliminates 2 years of data obtained by the Donnally study. Including the Donnally study eliminates all 2-level fusion data from the Yang study. Maintaining the standard set by the Kazarian 2022 and Patel 2022 Meta-Analyses, we have elected to use the Yang data and not use the Donnally data for our Meta-Analyses [5,6].

Seavey et al. was a retrospective study that utilized the Military Health Systems Data Repository from 2009 to 14 to evaluate preoperative lumbar epidural steroid injections association with postoperative SSI related to 1-2 level lumbar decompression surgery. Lumbar surgeries were identified via CPT codes 63005, 63030, 63047, 63056, and 63005. Lumbar epidural steroid injections were identified via CPT codes 023T, 64483, and 64484. Patients who received facet interventions were excluded. Surgical site infections were defined by ICD-9 codes 998.51 and 998.59 within 90 days of the index lumbar decompression surgery. Surgeries that preoperatively involved infection or tumor were excluded. They found no statistically significant risk associated with preoperative lumbar epidural steroid injections at 0-30 days, 31-90 days, 0-90 days, 91-180 days, 181-365 days, or >365 days. Given the differences in outcomes from this study relative to previously published studies, specifically Yang et al., they ran an additional cohort analysis that included only patients >65 years old to isolate a Medicare-eligible population. There was an increase in infection rates in the >65-year-old population who received lumbar epidural steroid injections compared to those who had not, but the difference was not statistically significant [10].

Pisano et al. was a retrospective study that utilized the Military Health Services Database from 2009 to 14 to evaluate preoperative ESI association with postoperative SSI related to lumbar fusion surgery. Lumbar surgeries were identified via CPT codes. To differentiate between the 2, ESI and facet interventions were identified via CPT codes, including the raw data. SSI was defined by ICD codes. They found no statistically significant risk associated with preoperative ESI and/or facet interventions with preoperative SSI at 0-30 days, 31-90 days, 91-180 days, 181-365 days, or >365 days [14].

Koltsov et al. used the IBM MarketScan Database from 2007 to 15 to retrospectively evaluate all patients who underwent lumbar spine surgery, both fusion and nonfusion, for lumbar disc herniation or lumbar spinal stenosis. The defined ESI using CPT codes. They then evaluated those with a preoperative epidural steroid injection at 0-30 days, 31-60 days, 61-90 days, and 91-365 days to matched controls who had not undergone preoperative ESI. There was no statistically significant difference at any measured time in postoperative surgical site infections when comparing those who received a preoperative ESI to those who did not [1].

Wadhwa et al. was a retrospective study that utilized the IBM MarketShare Database from 2007 to 16 to evaluate preoperative ESI association with postoperative SSI as it relates to cervical spine surgery for cervical degenerative disorders. They excluded patients <18 years old, surgeries related to tumors, or surgeries related to trauma. They isolated the cohort via ICD-9 identification of those with cervical spinal degenerative disorders and then cross-referenced those codes with the CPT codes for 1-2 level cervical spine surgeries. They defined ESI using multiple CPT codes which were not specific to an ESI injection. They included patients in their exposure cohort who underwent cervical epidural steroid injections, lumbar epidural steroid injections, epidural catheter placement, and facet interventions. They evaluated preoperative injections at 3, 6, 12, 18, and 24 months. The evaluated postoperative SSI was determined by ICD codes for reoperation within 90 days from the index surgery. They then matched controls to the respective measured time periods in the injection cohort. They detected no statistically significant difference between those who received a preoperative spinal injection at any point and SSI within 90 days of index cervical spine surgery, except for a statistically significant difference identified among those receiving preoperative injections at 91-180 days before cervical spine surgery. However, this study did not draw its conclusion from epidural steroid injections alone. Therefore, we cannot definitively conclude that any specific injection is associated with the difference observed at the 91-180-day time point [16].

### Prospective cohort studies

Farshad et al. prospectively examined patients who underwent lumbar spinal decompression with or without fusion surgery in the Swiss Lumbar Stenosis Outcome Study, a multicenter cohort study of patients with symptomatic lumbar spinal stenosis from hospitals in Switzerland. The study looked at patients who developed SSI versus matched controls who did not. They then reviewed whether those patients had or had not received a spinal injection before their operation. The study does not delineate between epidural steroid injections, facet joint interventions, or other spinal injections. There was no statistically significant difference in SSI risk observed between those with a preoperative spinal injection and those without a preoperative spinal injection [21].

Li et al. prospectively examined patients from 2015 to 2019 who underwent posterior lumbar fusion at a single center in China. They compared those who did and did not receive a preoperative ESI before their operation within 0-30 days and >30 days from their operation. In the 0-30 day injection group, 3.5 +/- 1.0 levels were fused. In the > 30-day day injection group, 3.3 +/- .9 levels were fused. In the control group, 3.2 +/- .9 levels were fused. It is unclear from the publication how patients were selected for epidural injections. All patients who received a preoperative epidural steroid injection matriculated to surgical intervention. Furthermore, the article reports that some epidural injections were performed with steroids while others were performed with lidocaine alone. There is no explanation for why some might get steroids while others lidocaine alone. The published data does allow for the delineation of those who got an epidural steroid injection and those who received an epidural lidocaine injection. Of the studies reviewed, this study had the highest infection rate, with a control rate of 3.5% and a < 30-day ESI rate of 10.5%. They produced a statistically significant association between preoperative ESI when given <30 days from surgery and postoperative SSI. The association did not exist in the <30 days group when no steroid was used in the epidural injection. However, no association beyond that time point was found [18] (Table 2).

**Table 2:** Statistical summary.

Author Year, Study Type	Injection SSI	Injection No SSI	Control SSI	Control No SSI	Statistical Commentary
Yang 2015, Retrospective	0-30 days: 38 31-90 days: 68 91-180 days: 58 181 - 365 days: 32 Total: 196	0-30 days: 2,223 31-90 days: 5,629 91-180 days: 6,959 181 - 365 days: 3,924 Total: 18,735	0-30 days: 190 31-90 days: 186 91-180 days: 154 181 - 365 days: 116 Total: 646	0-30 days: 36,396 31-90 days: 27,762 91-180 days: 23,082 181 - 365 days: 18,659 Total: 105,899	The study did not publish the actual infection # in each subcohort from the control group. They did publish the total amount in each subcohort control group. Therefore, we took that value, used the published data from the exposure group, the published OR, and worked backward to estimate the # infected in each subcohort.
Zusman 2015, Retrospective	3	114	1	171	The total number of control patients (163) in the Abstract, Table 1, and Table 2 of control patients from the manuscript do not match the number of control patients described (172). We elected to use the 172 figure, given that the percentages reported align with that figure when calculated within the manuscript.
Hartveldt 2016, Retrospective	0-30 days: 5 31-90 days: 15 0-90 days: 20	0-30 days: 285 31-90 days: 746 0-90 days: 1,031	0-30 days: 129 31-90 days: 119 0-90 days: 115	0-30 days: 4,892 31-90 days: 4,431 0-90 days: 4,251	There is 100% agreement between the raw data, published unadjusted OR, and manually produced unadjusted OR. We could not reconcile why the summation of the 0-30 and 30-90 groups differed from that of the 0-90 group. Despite this incongruency, we elected to use the data as published.
Cancienne 2017, Retrospective	Total: 122 PCF 0-90 days: 16 91-180 days: 19 180-365 days: 14 Total: 49 ACDF 0-90 days: 34 91-180 days: 22 180-365 days: 17 Total: 73	Total: 14,680 PCF 0-90 days: 386 91-180 days: 567 180-365 days: 615 Total: 1,568 ACDF 0-90 days: 4,320 91-180 days: 5,161 180-365 days: 3,631 Total: 13,112	Total: 2.671 PCF 1,305 ACDF 1,366	Total: 302,931 PCF 59,948 ACDF 240,312	We cannot reproduce the published subcohort OR's using the published data.
Seavey 2017, Retrospective	0-30 days: 1 31-90 days: 5 91-180 days: 3 181-365 days: 1 >365 days: 0 Total: 10	0-30 days: 166 31-90 days: 313 91-180 days: 199 181-365 days: 88 >365 days: 71 Total: 837	43	5,645	The control (43/5,688) was not divided by timing subcohort, yet appears to have been used to produce the published subcohort OR.
Singla 2017, Retrospective	0-30 days:66 30-90 days: 120 90-180 days: 136 Total: 322	0-30 days: 1,633 30-90 days: 5,371 90-180 days: 10,357 Total: 17,361	1,089	69,768	The control (1,089/70,857) was not divided by timing subcohort yet appears to have been used as the control for each timing subcohort.
Ozturk 2018, Retrospective	0-30 days: 0 31-90 days: 0 91-180 days: 0 181-365 days: 0 >365 days: 0 Total: 0	0-30 days: 9 31-90 days: 12 91-180 days: 2 181-365 days: 3 >365 days: 6 Total: 31	0	35	Raw data is not controlled for time in the control group. Therefore, the control data is not usable for subcohort calculations.
Donnally 2018, Retrospective	0-30 days: 15 31-90 days: 51 91-180 days: 58 Total: 124	0-30 days: 740 31-90 days: 3,158 91-180 days: 4,068 Total: 7,966	Not published	8,090	The study reports a single control group for “lumbar decompression with no 6-month LESI history.” We are unable to reconcile the published ORs using the published data.
Farshad 2018, Prospective	4	14	2	5	
Pisano 2019, Retrospective	0 - 30 days: 0 31 - 90 days: 0 91 - 365 days: 5 Total: 5	0 - 30 days: 22 31 - 90 days: 85 91 - 365 days: 241 Total: 348	43	2,748	Control data was not controlled for time yet appears to have been used for subcohort comparisons. Using the 43/2,791 figure, which is not controlled for time, the 91-180 days, 181-365 days, and >365 days ORs seem to be close enough to reasonably conclude they used the 43/2,791 figure with some statistical adjustments.
Koltsov 2020, Retrospective	0 - 30 days: 504 31- 60 days: 622 61- 90 days: 451 91 - 365 days: 1,136 Total: 2,713	0 - 30 days: 23,562 31- 60 days: 25,470 61- 90 days: 17,817 91 - 365 days: 40,448 Total: 107,297	0 - 30 days: 490 31- 60 days: 601 61- 90 days: 471 91 - 365 days: 1,156 Total: 2,718	0 - 30 days: 23,576 31- 60 days: 25,491 61- 90 days: 17,797 91 - 365 days: 40,428 Total: 107,292	None
Li 2020, Prospective	0-30 days: 11 31+ days: 8	0-30 days: 94 31+ days: 132	81	2,231	The study does not appear to have produced unique time-matched subcohorts for their control.
Kreitz 2020, Retrospective	Decompression: 0-30: 7 31-90: 8 >90:15 Total: 30 Fusion: 0-30: 5 31-90: 7 >90: 25 Total: 37	Decompression: 0-30: 501 31-90: 1,244 >90:1,182 Total: 2,927 Fusion: 0-30: 82 31-90: 450 >90: 814 Total: 1,346	Decompression:67 Fusion: 63	Decompression: 6,879 Fusion: 3,662	The control data was not controlled for each subcohort. We could not reconcile the difference in OR between the published OR and our manually calculated figure.
Wadhwa 2021, Retrospective	0-90 days: 73 0-180 days: 107 0-365 days: 119 0-545 days:129 0-730+ days: 132 Total: 132	0-90 days: 16,641 0-180 days: 23,079 0-365 days: 27,069 0-545 days: 28,681 0-730+ days: 29,831 Total: 29,831	0-90 days: 206 0-180 days: 72 0-365 days: 125 0-545 days:111 0-730+ days: 127	0-90 days: 49,936 0-180 days: 23,114 0-365 days: 27,063 0-545 days: 28,699 0-730+ days: 29,836	None
Shakya 2022, Retrospective	1	128	2	184	None

**Table 3:** ICD/CPT summary.

Code	Studies	Results
62310	Cancienne, Wadhwa	Injection(s), of diagnostic or therapeutic substance(s) (including anesthetic, antispasmodic, opioid, steroid, other solution), not including neurolytic substances, including needle or catheter placement, includes contrast for localization when performed, **epidural** or subarachnoid; cervical or thoracic [1].
62311	Kreitz, Hartveldt, Koltsov, Yang, Donnally, Singla, Wadhwa	Injection(s), of diagnostic or therapeutic substance(s) (including anesthetic, antispasmodic, opioid, steroid, other solution), not including neurolytic substances, including needle or catheter placement, includes contrast for localization when performed, **epidural** or subarachnoid; lumbar or sacral (caudal) [1]
62318	Wadhwa	Injection(s), including indwelling catheter placement, continuous infusion or intermittent bolus, of diagnostic or therapeutic substance(s) (including anesthetic, antispasmodic, opioid, steroid, other solution), not including neurolytic substances, includes contrast for localization when performed, **epidural** or subarachnoid; cervical or thoracic [1].
62319	Wadhwa	Injection(s), including indwelling catheter placement, continuous infusion or intermittent bolus, of diagnostic or therapeutic substance(s) (including anesthetic, antispasmodic, opioid, steroid, other solution), not including neurolytic substances, includes contrast for localization when performed, **epidural** or subarachnoid; lumbar or sacral (caudal) [1].
64470	Wadhwa	Injection, anesthetic agent and/or steroid, paravertebral facet joint or facet joint nerve; cervical or thoracic, single level [2].
64472	Wadhwa	Injection, anesthetic agent and/or steroid, paravertebral facet joint or facet joint nerve; cervical or thoracic, each additional level [2].
64475	Kreitz, Pisano, Wadhwa	Injection, anesthetic agent and/or steroid, paravertebral facet joint or facet joint nerve; lumbar or sacral, single level [2].
64476	Pisano, Wadhwa	Injection, anesthetic agent and/or steroid, paravertebral facet joint or facet joint nerve; lumbar or sacral, each additional level [2].
64479	Cancienne, Wadhwa	This procedure is the injection of an anesthetic agent and/or steroid in the form of a transforaminal epidural injection into a single level (either cervical or thoracic) [3].
64480	Wadhwa	This procedure is the injection of an anesthetic agent and/or steroid in the form of a transforaminal **epidural** injection in the cervical or thoracic region. The code applies to each additional level after the initial level [4].
64483	Kreitz, Hartveldt, Seavey, Koltsov, Pisano, Yang, Donnally, Singla, Wadhwa	This procedure is the injection of an anesthetic agent and/or steroid in the form of a transforaminal epidural injection into a single level (either lumbar or sacral) [5].
64484	Hartveldt, Seavey, Koltsov, Pisano, Wadhwa	This procedure is the injection of an anesthetic agent and/or steroid in the form of a transforaminal epidural injection in the lumbar or sacral region [6].
64493	Kreitz. Pisano	In this service, the provider injects a diagnostic or therapeutic agent into a facet joint, the joint connecting 2 spinal vertebrae together, at the lumbar or sacral level. Or he may perform the injection for nerves innervating that joint. He uses imaging guidance of either fluoroscopy or CT scan [7].
996.67	Kreitz, Cancienne, Donnally, Singla	Infection and inflammatory reaction due to other internal orthopedic device implant and graft [8].^(p67)^
996.69	Cancienne	Infection and inflammatory reaction due to other internal prosthetic device implant and graft [9].^(p69)^
998.12	Kreitz	Hematoma complicating a procedure [10].
998.31	Kreitz	Disruption of internal operation (surgical) wound [11].^(p31)^
998.32	Kreitz	Disruption of external operation (surgical) wound [12].^(p32)^
998.5	Cancienne, Yang, Donnally, Singla	Postoperative infection not elsewhere classified [13].^(p5)^
998.51	Seavey, Cancienne, Pisano, Yang, Donnally, Singla	Infected postoperative seroma [14].^(p51)^
998.59	Kreitz, Cancienne, Pisano, Yang, Donnally, Singla, Seavey	Other postoperative infection [15].
T81.31XA	Kreitz	Disruption of external operation (surgical) wound, not elsewhere classified, initial encounter [16].
T81.32XA	Kreitz	Disruption of internal operation (surgical) wound, not elsewhere classified, initial encounter [17].
T84.7XXA	Kreitz	Infection and inflammatory reaction due to other internal orthopedic prosthetic devices, implants and grafts, initial encounter [18].
0216T	Pisano	The provider injects a diagnostic or therapeutic agent under ultrasound guidance into a single lumbar or sacral paravertebral facet joint or the nerves that exit the joint [19].
0217T	Hartveldt, Pisano	The provider injects a diagnostic or therapeutic agent into an additional lumbar or sacral paravertebral facet joint or the nerves that exit the joint under ultrasound guidance [20].
0230T	Hartveldt, Seavey, Pisano	Injection(s), anesthetic agent and/or steroid, transforaminal **epidural**, with ultrasound guidance, lumbar or sacral; single level [21].
0231T	Hartveldt	Injection(s), anesthetic agent and/or steroid, transforaminal **epidural**, with ultrasound guidance, lumbar or sacral; each additional level [21].
20005	Cancienne, Yang, Donnally, Singla	Incision and drainage of soft tissue abscess, subfascial (ie., involves the soft tissue below the deep fascia) [22].
10180	Cancienne	The provider incises the area of infection and drains any fluid collection, with the help of surgical instruments [23].
21501	Cancienne	A provider performs an incision and drainage procedure in the deep tissues of the neck or chest to relieve pain and pressure from a pocket of blood or pus [24].
22015	Yang, Donnally, Singla	The provider incises and drains an abscess, or pocket of infection, in the deep tissues at the back of the lower spinal column to relieve pain and pressure [25].

1. Billing M. CPT CODE 62310, 62311 – Epidural injection | Medicare Payment, Reimbursement, CPT code, ICD, Denial Guidelines. Accessed November 19, 2022. https://medicarepaymentandreimbursement.com/2016/09/cpt-code-62310-62311-epidural-injection.html

2. aapc admin. Facet Joint Injections: Code with Precision. AAPC Knowledge Center. Published December 1, 2008. Accessed November 19, 2022. https://www.aapc.com/blog/24029-facet-joint-injections-code-with-precision/

3. CPT® Code 64479 - Introduction/Injection of Anesthetic Agent (Nerve Block), Diagnostic or Therapeutic Procedures on the Somatic Nerves - Codify by AAPC. Accessed November 19, 2022. https://www.aapc.com/codes/cpt-codes/64479

4. CPT® Code 64480 - Introduction/Injection of Anesthetic Agent (Nerve Block), Diagnostic or Therapeutic Procedures on the Somatic Nerves - Codify by AAPC. Accessed November 19, 2022. https://www.aapc.com/codes/cpt-codes/64480

5. CPT® Code 64483 - Introduction/Injection of Anesthetic Agent (Nerve Block), Diagnostic or Therapeutic Procedures on the Somatic Nerves - Codify by AAPC. Accessed November 19, 2022. https://www.aapc.com/codes/cpt-codes/64483

6. CPT® Code 64484 - Introduction/Injection of Anesthetic Agent (Nerve Block), Diagnostic or Therapeutic Procedures on the Somatic Nerves - Codify by AAPC. Accessed November 19, 2022. https://www.aapc.com/codes/cpt-codes/64484

7. CPT® Code 64493 - Introduction/Injection of Anesthetic Agent (Nerve Block), Diagnostic or Therapeutic Procedures on the Paravertebral Spinal Nerves and Branches - Codify by AAPC. Accessed November 19, 2022. https://www.aapc.com/codes/cpt-codes/64493

8. ICD-9 Code 996.67 -Infection and inflammatory reaction due to other internal orthopedic device implant and graft- Codify by AAPC. Accessed November 19, 2022. https://www.aapc.com/codes/icd9-codes/996.67

9. ICD-9 Code 996.69 -Infection and inflammatory reaction due to other internal prosthetic device implant and graft- Codify by AAPC. Accessed November 19, 2022. https://www.aapc.com/codes/icd9-codes/996.69

10. ICD-9 Code 998.12 -Hematoma complicating a procedure- Codify by AAPC. Accessed November 19, 2022. https://www.aapc.com/codes/icd9-codes/998.12

11. ICD-9 Code 998.31 -Disruption of internal operation (surgical) wound- Codify by AAPC. Accessed November 19, 2022. https://www.aapc.com/codes/icd9-codes/998.31

12. ICD-9 Code 998.32 -Disruption of external operation (surgical) wound- Codify by AAPC. Accessed November 19, 2022. https://www.aapc.com/codes/icd9-codes/998.32

13. ICD-9 Code 998.5 -Postoperative infection not elsewhere classified- Codify by AAPC. Accessed November 19, 2022. https://www.aapc.com/codes/icd9-codes/998.5

14. ICD-9 Code 998.51 -Infected postoperative seroma- Codify by AAPC. Accessed November 19, 2022. https://www.aapc.com/codes/icd9-codes/998.51

15. ICD-9 Code 998.59 -Other postoperative infection- Codify by AAPC. Accessed November 19, 2022. https://www.aapc.com/codes/icd9-codes/998.59

16. ICD-10-CM Code for Disruption of external operation (surgical) wound, not elsewhere classified, initial encounter T81.31XA. https://www.aapc.com/codes/icd-10-codes/T81.31XA

17. ICD-10 Code for Disruption of internal operation (surgical) wound, not elsewhere classified, initial encounter- T81.32XA- Codify by AAPC. Accessed November 19, 2022. https://www.aapc.com/codes/icd-10-codes/T81.32XA

18. ICD-10 Code for Infection and inflammatory reaction due to other internal orthopedic prosthetic devices, implants and grafts, initial encounter- T84.7XXA- Codify by AAPC. Accessed November 19, 2022. https://www.aapc.com/codes/icd-10-codes/T84.7XXA

19. CPT® Code 0216T - Various Services - Category III Codes - Codify by AAPC. Accessed November 19, 2022. https://www.aapc.com/codes/cpt-codes/0216T

20. CPT® Code 0217T - Various Services - Category III Codes - Codify by AAPC. Accessed November 19, 2022. https://www.aapc.com/codes/cpt-codes/0217T

21. aapc admin. New Codes, New Rates in July for ASCs. AAPC Knowledge Center. Published July 2, 2010. Accessed November 19, 2022. https://www.aapc.com/blog/6856-new-codes-new-rates-in-july-for-ascs/

22. Verhovshek J. Coding Abscess Procedures. AAPC Knowledge Center. Published December 19, 2016. Accessed November 19, 2022. https://www.aapc.com/blog/37219-coding-abscess-procedures/

23. CPT® Code 10180 - Incision and Drainage Procedures on the Skin, Subcutaneous and Accessory Structures - Codify by AAPC. Accessed November 19, 2022. https://www.aapc.com/codes/cpt-codes/10180

24. CPT® Code 21501 - Incision Procedures on the Neck (Soft Tissues) and Thorax - Codify by AAPC. Accessed November 19, 2022. https://www.aapc.com/codes/cpt-codes/21501

25. CPT® Code 22015 - Incision Procedures on the Spine (Vertebral Column) - Codify by AAPC. Accessed November 19, 2022. https://www.aapc.com/codes/cpt-codes/22015

## Cohort and subcohort forest plots and odds ratios

I. ESI Only Studies Not Controlled for Time

Eight studies were included in the meta-analysis of postoperative infection risk following exclusively epidural steroid injections with no control for time. A total of 2.12% (3,383/159,295) of patients who underwent a preoperative epidural steroid injection experienced a postoperative infection compared to 1.23% (7,250/591,314) of controls. This represented a statistically significant increase in surgical site infection risk (OR= 1.08, 95% CI 1.03-1.13, p<.00001; heterogeneity: I2= 88%). The Number Needed to Harm (NNH) is 111 patients.

II. ESI only studies within 0-30 days from lumbar surgery

Eight studies were included in the meta-analysis for postoperative infection risk stratified to include all lumbar studies controlled exclusively for epidural steroid injections received 30 days or less prior to lumbar surgery. A total of 2.23% (637/28568) of patients experienced postoperative infection, as compared to 1.41% (2,195/155797) of controls, which did constitute a statistically significant difference (OR= 1.28, 95% CI 1.15-1.43, p<.00001); heterogeneity: I^2^= 91%). The Number Needed to Harm (NNH) is 95 patients.

III. ESI only studies within 31-90 days from lumbar surgery

Seven studies were included in the meta-analysis for postoperative infection risk stratified to include all lumbar studies controlled exclusively for epidural steroid injections received 31-90 days prior to lumbar surgery. A total of 2.27% (1,296/57,125) of patients experienced postoperative infection, as compared to 1.63% (2,682/164,183) of controls, which did constitute a statistically significant difference (OR=1.08, 95% CI 1.00-1.16, p<.00001); heterogeneity: I2= 78%). The NNH is 102 patients.

IV. All studies not controlled for time

Fourteen studies were included in the meta-analysis of postoperative infection risk following spinal injections with no control for time. Studies that included non-ESI-specific data were included in this figure. A total of 1.85% (3,613/194796) of patients who underwent a preoperative epidural steroid injection experienced a postoperative infection compared to 1.2% (7,668/638866) of controls. This represented a statistically significant increase in surgical site infection risk (OR= 1.08, 95% CI 1.03-1.12, p<.00001; heterogeneity: I2= 79%).

V. All cervical studies only not controlled for time

2 studies were included in the meta-analysis of postoperative infection risk stratified out to include all cervical studies with no control for time. This meta-analysis included studies that included spinal injections beyond epidural steroid injections, namely facet interventions. A total of 0.57% (254/44,511) of patients who underwent a preoperative injection experienced postoperative infection compared to 0.85% (2,798/330,096) of controls. This represented no statistically significant association with surgical site infection risk (OR= 1.09, 95% CI 0.94-1.27, p=.10; heterogeneity: I2= 57%).

VI. Additional subgroup analyses

Five studies were included in the meta-analysis for postoperative infection risk stratified to include studies that exclusively evaluated lumbar fusion with no control for time. This meta-analysis included studies that included spinal injections beyond epidural steroid injections, namely facet interventions. A total of 1.99% (386/19,390) of patients experienced postoperative SSI, as compared to 1.63% (1,277/78,571) of controls. This represented a statistically significant increase in surgical site infection risk (OR= 1.26, 95% CI 1.12-1.41, p<.0001; heterogeneity: I2=60%) [7,11,14,15,18].

Three studies were included in the meta-analysis for postoperative infection risk stratified to include studies that exclusively evaluated lumbar decompression with no control for time. This meta-analysis included studies that included spinal injections beyond epidural steroid injections, namely facet interventions. A total of 1.00% (226/22,499) of patients experienced postoperative SSI compared to 0.63% (756/118,423) of controls. This represented a statistically significant increase in surgical site infection risk (OR= 1.55, 95% CI 1.33-1.80, p<.00001; heterogeneity: I2= 82%) [9,10,15].

Three studies were included in the meta-analysis for postoperative infection risk stratified to include only studies from the Medicare pearlDiver database with no control for time. This meta-analysis included studies that included spinal injections beyond epidural steroid injections, namely facet interventions. A total of 1.26% (640/50,776) of patients experienced postoperative infection compared to 0.93% (4,406/475,927) of controls. This represented a statistically significant increase in surgical site infection risk (OR= 1.30, 95% CI 1.19-1.42, p<.00001; heterogeneity: I2= 85%) [9,11,19].

Four studies were included in the meta-analysis for postoperative infection risk stratified to include only studies from the IBM MarketScan and Military Health Services databases with no control for time. This meta-analysis included studies that included spinal injections beyond epidural steroid injections, namely facet interventions. A total of 2.07% (2,860/138,440) of patients experienced postoperative infection, as compared to 2.01% (2,931/145,648) of controls, which did not constitute a statistically significant increase in surgical site infection risk (OR= 1.00, 95% CI 0.95-1.06, p=.94; heterogeneity: I2= 0%) [1,10,14,16].

Eleven studies were included in the meta-analysis for postoperative infection risk stratified to include only studies from the non-Medicare PearlDiver database studies with no control for time. This meta-analysis included studies that included spinal injections beyond epidural steroid injections, namely facet interventions. A total of 2.06% (2,963/144,152) of patients experienced postoperative infection compared to 2.00% (3,262/17,743) of controls. This did not represent a statistically significant increase in surgical site infection risk (OR= 1.01, 95% CI 0.96-1.06, p=.010; heterogeneity: I2= 57%) [1,7,8,10,[13], [14], [15], [16], [17], [18],^21^].

## Odds ratio summary

**Table:** 

ESI only not controlled for time	1.08, 95% CI 1.03–1.13
**ESI only ≤ 30 days before lumbar surgery**	**1.28, 95% CI 1.15–1.43**
ESI only 31-90 days before lumbar surgery	1.08, 95% CI 1.00–1.16
ESI only >90 days before lumbar surgery	1.07, 95% CI 1.00–1.15
**All spinal injections not controlled for time**	**1.08, 95% CI 1.03–1.12**
All spinal injections cervical studies only not controlled for time	1.09, 95% CI 0.94–1.27
**All spinal injections lumbar fusion only not controlled for time**	**1.26, 95% CI 1.12–1.41**
**All spinal injections lumbar decompression only not controlled for time**	**1.55, 95% CI 1.33–1.80**
**All spinal injections only Medicare PearlDiver Database not controlled for time**	**1.30, 95% CI 1.19–1.42**
All spinal injections only Military Health Services and IBM MarketScan Database not controlled for time	1.00, 95% CI 0.95–1.06
All spinal injections not controlled for time excluding PearlDiver Database studies	1.01, 95% CI 0.96–1.06

Bolded for statistical significance.

## GRADE assessment of the evidence regarding the association between epidural steroids and postspinal surgery surgical site infections

When applying GRADE, the resulting body of evidence is assigned a “moderate” GRADE quality of evidence rating. There were no disagreements that required third-party intervention. This rating is attributed to the variety of definitions of surgical site infection and the variety in definitions of what type of injection qualified as an “epidural steroid injection.” Only 1 study identified the epidural technique, medication, and dose when discussing an epidural steroid injection. There was an inability to assess the specifics regarding the instrumentation used for fusion surgeries and the accuracy of the databases accessed for many of the large retrospective studies [22]. We are moderately confident in the estimate based on the published literature. However, there is a possibility that prospective studies may yield different results.

## Discussion

There appears to be a statistically significant association between preoperative ESI and postoperative lumbar spine SSI. The association was statistically strongest at ESI 0-30 days from surgery, less so at ESI 31-90 days from lumbar spinal surgery, and no longer statistically significant when the ESI was given >90 days from the lumbar spinal surgery. These results suggest a timing association between when the ESI is given preoperatively and preoperative ESI.

The odds ratios are close enough to 1.0 regardless of stratification by surgery type, the database used, or specific injection that clinically the effect size is “small” or “weak” [23,24]. For some providers, such an effect size may be irrelevant in decision-making.

There were 2 previously published Meta-Analyses by Kazarian and Patel. Kazarian recommended against all corticosteroid injections within 1 month of any spinal surgery. It should be noted that all corticosteroid injections are not synonymous with epidural steroid injections. While our study did not address “all corticosteroid injections,” some of our meta-analyses, which included non-ESI spine injections, found that there was a small, but likely clinically irrelevant, association between preoperative spinal injections and postoperative SSI regardless of time given prior to surgery (1.08, 95% CI 1.03-1.12).

However, in Kazarian's “leave-out” meta-analysis, they eliminated the results of Singla and Yang. The exclusion eliminated the association between CSI 0-30 days before surgery and postoperative infection, suggesting a weighted bias from those 2 studies. Kazarian concluded that an association between preoperative CSI 0-30 days before surgery and postoperative infection hinged solely on including Singla and Yang.

A key facet of the Singla and Yang studies is that they share the same data source, Medicare PearlDiver. We also found that when eliminating the Medicare PearlDiver database from our meta-analyses, a statistically significant association was no longer present, small as it may have been. Our analysis further strengthens the signal that the Medicare PearlDiver database may uniquely capture a patient population more at risk for preoperative SSI than other sources.

### Limitations of the evidence included in the review

Our conclusions are limited as the data is >99% retrospective and lacks specificity regarding the injections performed to draw more helpful conclusions to guide clinical decision-making. Only 1 study identified the type of steroid, amount of injectate, injection approach, and localization of injectate. Any of these factors may affect the potential risk passed to patients. Interestingly, 3 studies exclusively reviewed the use of TFESI [10,13,17]. The pooled data from those studies, which includes decompression and discectomy patients, produces no statistically significant difference (1.44 [0.74-2.79] p=.2815) in preoperative surgical site infection, which raises the question regarding whether technique plays a role.

The Medicare PearlDiver Database is a private national database comprising 41 billion patient records from commercial insurance, government claims, and other sources. The database is restricted to coding and billing of patients 65 or older and thus may misrepresent certain aspects of care given regional variability in coverage. The database evaluates potential associations between variables but should look to prospective studies to determine causal relationships [25,26].

The IBM MarketScan database comprises commercial claims from inpatient, outpatient, and pharmaceutical claims of over 75 million employees, retirees, and dependents. A substantial portion of the American population is covered by employer-sponsored insurance and thus represented in this database [16].

The Military Health Services Data Repository is made up of over 9 million military and civilian patients from the age of 18-64. While a pediatric population is included as well, for our database reviews, this portion of the database was not queried per the study's methodology [27].

In our meta-analysis, only the Medicare PearlDiver database detected a statistically significant risk associated with preoperative epidural steroid injection. Is this finding due to a confounding variable reporting error, or is it a real independent risk factor unique to the Medicare PearlDiver patient population? Database reviews are limited by the accuracy of coding, the populations from which they are drawn, and the specificity of the coding. Their limitations are well documented [22,28,29].

### Implications for practice, policy, and future research

This study is warranted because, in 2022, there were 2 different meta-analyses, which used 2 different sets of data and reached 2 different conclusions, with each suggesting changes to practice habits. Our effort is now the third entry, but it uses more studies and raw data and attempts to inform readers regarding the heterogeneity of studies published to date to best inform clinical decision-making.

While we also concluded a time-dependent statistically significant association between preoperative ESI and postoperative SSI, we cannot comment on the temporal relationship based on the type of spinal surgery (decompression vs fusion) based on statistical issues regarding controlling for time described in Table 1. Based on the available evidence, there is a time-dependent statistically significant association between preoperative ESI and postoperative SSI. However, the data does not elevate to a level that should limit ESI use due to a risk passed along to the patient.

**Table 1:** Included studies.

Author Year, Study Type	Type of Surgery	Sample Source; Years	Total Sample	Population Characteristics	ESI Definition	SSI Definition	Risk of Bias
Kreitz 2020, Retrospective	All elective lumbar fusion or decompression for radiculopathy and/or spinal stenosis; 63030, 63047, 22612 with minimum 90 day follow up	Single Site, Philadelphia, PA; 2000-17	Total: 15,001 Decompression: 9,903 Fusion: 5,108	All elective lumbar spine procedures performed for a diagnosis of lumbar radiculopathy and/or spinal stenosis with minimum 90 days follow-up. Excluded: trauma, pre-existing infection, tumor, and revisions	62311, 64475, 64483, 64493	Post-operative SSI requiring reoperation <90 days; 996.67, 998.12, 998.31, 998.32, 998.59, T81.31XA, T81.32XA, T84.7XXA	64475 and 64493 are CPT codes for facet injections, not ESI. Therefore, the results of the study may be confounded by the inclusion of procedures which do not appear to have been intended to be included.
Hartveldt 2016, Retrospective	Single or multilevel lumbar laminectomy with or without arthrodesis; 22612, 22558, 22630, 22808, 22810 for arthrodesis; 63047, 63030, 22630, 63005, 63017 for laminectomy	Multi Site, Boston, MA; 2005-15	5,311	18 y/o with at least 90 days of clinical follow-up. Excluded: tumor, fracture, trauma, pseudoarthrosis, pre-existing infection	62311, 0217T, 0230T, 0231T, 64483, and 64484	Postoperative SSI as symptoms clinically consistent requiring an incision and drainage intervention performed in the operating room	Underpowered with 5,311 participants. “To detect this difference…we would have needed a sample size of 30,214 patients.” 0217T is a CPT code for facet injections, not LESI. Therefore, the results of the study may be confounded by the inclusion of procedures which do not appear to have been intended to be included.
Zusman 2015, Retrospective	Thoracic and/or lumbar arthrodesis	Single Site, Portland, OR; 2007-10	289	Elective thoracic and/or lumbar arthrodesis who had completed pre-operative and 90-day postoperative outcome testing (SF-12, ODI). Excluded: trauma, tumor, infection.	Patient-reported preoperative spinal injection	Surgical wound complications included hematoma, seroma, and infection, requiring an unplanned reoperation within 30 days of index surgery	Defining ESI as “patient-reported spinal injection” likely induces recall bias, which may impact the results. Unclear when the injection was given preoperatively or what injection was specifically given. Study does not separate between thoracic surgical intervention and lumbar surgical intervention
Ozturk 2018, Retrospective	Microdiscectomy	Single Site, Turkey; 2011-15	66	Pts who had undergone unilateral, single-level lumbar microdiscectomy due to extruded or sequestrated lumbar discs Excluded: BMI >30, Diabetes, Renal Failure, ischemic heart or cerebrovascular disease	Transforaminal anterior epidural steroid injection (TAESI); 80 mg triamcinolone + 3 mL .5% bupivacaine	No infection definition	No infections reported in epidural or control groups Only study that defines ESI contents
Seavey 2017, Retrospective	1-2 Level lumbar laminectomy/decompression: 63005, 63030, 63047, 63056	Military Health System Data Repository; 2009-14	6,535	Pts w/ Lumbar ESI prior to single-level lumbar decompression Excluded: Multi-level decompressions (except for 63005), revision surgeries, prior infection, tumor; those who had prior facet injections	0230T, 64483, 64484	SSI within 90 days post op ICD-9 codes :998.51, 998.59	Database study with inherent accuracy concerns. Unable to rule out bias in patient selection or surgical indication. Unknown data on the number of previous LESI's, steroid dose, level of injections, type of steroid, additional steroid usage, surgical complexity.
Cancienne 2017, Retrospective	ACDF (22554, 22551, 22585, 63076, ICD-9 81.02) PCF (22590, 22600, ICD-9 81.03)	Medicare PearlDiver Database; 2005-12	Total: 317,733 ACDF: 254,863 PCF: 62,870	Patients w/ PCF or ACDF divided by time from CESI and matched with controls in the same timing sub-cohorts Excluded: Fusions above C2 (22590, 22595), revision surgeries	64479, 62310	Post op infection within 90 days CPT: 20005, 10180, 21501 ICD-9: 998.5, 998.51, 998.59, 996.67, 996.69	Database study with inherent accuracy concerns. Unable to rule out bias in patient selection or surgical indication. Unknown data on the number of previous CESI's, steroid dose, level of injections, type of steroid, additional steroid usage, surgical complexity, instrumentation specifics.
Koltsov 2020, Retrospective	Lumbar decompression, fusion, discectomy. CPT/ICD codes not published	IBM MarketScan Database; 2007-15	220,020	Patients with disc herniation or stenosis, or both who underwent lumbar decompression, fusion or discectomy. Excluded: reoperations, neoplasms, intraspinal abscesses, osteomyelitis, discitis, fracture, dislocation, vehicular accidents, inflammatory spondyloarthropathies, rheumatoid arthritis	64483, 64484, 62311; 0-30 days, 31-60, 61-90, 91-365 days	Post op infection within 90 days from index using “codes published previously.”	They did not separate results by fusion or non-fusion surgical interventions. Therefore, the data was not applicable for Meta-Analyses sub-cohorts which looked exclusively at fusion or non-fusion. Database study with inherent accuracy concerns. Unable to rule out bias in patient selection or surgical indication. Unknown data on the number of previous LESI's, steroid dose, level of injections, type of steroid, additional steroid usage, surgical complexity, instrumentation specifics.
Li 2020, Prospective	Posterior lumbar multi-level fusion for degenerative disc disease	Single Site, China; 2015-19	2,557	Diagnosis of lumbar disc herniation or another degenerative lumbar spine disease con- firmed by radiological examination in the setting of chronic low back pain associated with radicular symptoms >6 months; and participation in medical therapy or physical rehabilitation for >3 months without improvement Excluded: h/o minimally invasive procedures not performed in the OR, lumbar surgery, spinal infection, those who did not follow up	Lumbar transforaminal epidural injection of lidocaine with and without steroid	Surgical site infection related to the operation.	3+ levels of fusion on avg Unclear injection selection method. Moreover, all patients who were injected eventually had surgery. Some injections had steroids, others did not; it is not clear on how this decision was made
Pisano 2019, Retrospective	Lumbar arthrodesis 0195T, 22533, 22558, 22612, 22630, 22633	Military Health System Data Repository; 2009-14	3,139	Patients who have undergone lumbar spine surgery with and without lumbar corticosteroid injection before (facet and epidural injections included)	Lumbar ESI- 0230T, 64483, 64484; Lumbar Facet Injection- 0216T, 0217T, 64475, 64476, 64493	ICD-9 codes 998.51 and 998.59	Despite including facet interventions, published raw data allows for that data to be excluded. Database study with inherent accuracy concerns. Unable to rule out bias in patient selection or surgical indication. Unknown data on the number of previous LESI's, steroid dose, level of injections, type of steroid, additional steroid usage, surgical complexity.
Yang 2015, Retrospective	1-2 level lumbar decompression; 63005, 63030, 63047	Medicare PearlDiver Database; 2005-12	125,476	Medicare patients over age 65 who had a lumbar epidural steroid injection within 1 year of 1-2 level lumbar decompression Excluded: Multi-level decompressions (except 63,005), revision surgeries	64,483, 62,311	SSI within 90 days of index surgery; ICD-9: 998.5, 998.51, 998.59; CPT 20,005, 22,015	Database study with inherent accuracy concerns. Unable to rule out bias in patient selection or surgical indication. Unknown data on the number of previous LESI's, steroid dose, level of injections, type of steroid, additional steroid usage, surgical complexity.
Donnally 2018, Retrospective	1 level decompression; 63,030, 63,047	Medicare PearlDiver Database; 2005-14	16,180	Medicare patients over age 65 who had a lumbar epidural steroid injection within 1 year of 1-level lumbar decompression Excluded: fusion, revision surgeries	64,483, 62,311	SSI within 90 days of index surgery; ICD-9: 998.5, 998.51, 998.59; 996.67; CPT 20,005, 22,015	Database study with inherent accuracy concerns. Unable to rule out bias in patient selection or surgical indication. Unknown data on the number of previous LESI's, steroid dose, level of injections, type of steroid, additional steroid usage, surgical complexity.
Singla 2017, Retrospective	1-2 level posterior lumbar spinal fusion; 22,612,22,614,22,633,22,630	Medicare PearlDiver Database; 2005-12	88,540	Medicare patients over age 65 who had a lumbar epidural steroid injection within 1 year of 1-2 level lumbar fusion Excluded: revision surgeries	64,483, 62,311	SSI within 90 days of index surgery; ICD-9: 998.5, 998.51, 998.59, 996.67; CPT 20,005, 22,015	Database study with inherent accuracy concerns. Unable to rule out bias in patient selection or surgical indication. Unknown data on the number of previous LESI's, steroid dose, level of injections, type of steroid, additional steroid usage, surgical complexity, instrumentation specifics.
Wadhwa 2021, Retrospective	CPT for 1-2 level cervical spine surgery: 22,551 22,552 22,554 22,585 22,600 22,614 63,001 63,015 63,020 63,035 63,040 63,043 63,045 63,048 63,050 6,305 63,075 63,076 63,081 63,082	IBM MarketScan Database; 2007-16	59,926	Patients who had cervical degenerative disease and cervical surgery; ICD 9 for cervical degenerative disease 722.0, 722.4, 723.0-723.5, 721.0, 721.1, 722.71, 722.91, and cervical myelopathy 721.1, 722.71 Exclude: <18 y/o, >2 level procedures, tumor or trauma	62,310, 62,311, 64,479, 64,480, 64,483, 64,484, 64,470, 64,472, 64,475, 64,476, 62,318, 62,319	Surgical site infection is based on re-operation within 90 days and ICD coding which was not listed in the published supplement	Injections included, but did not stratify for, cervical ESI, lumbar ESI, epidural catheter placement, and facet interventions. Database study with inherent accuracy concerns. Unable to rule out bias in patient selection or surgical indication. Unknown data on the number of previous LESI's, steroid dose, level of injections, type of steroid, additional steroid usage, surgical complexity, instrumentation type.
Shakya 2022, Retrospective	Lumbar discectomy	Single Center, India; 2017-20	315	21-65 y/o + single-level disc herniation of lumbar spine + at least follow up beyond 6 months + operated via minimally invasive approach Excluded: trauma, tumor, infection, revision, fusion, concomitant cervical spine pathology, spondyloarthropathies, ILESI or caudal	TFESI only	Does not define surgical site infection, but reports that infection was obtained by review of hospital records	They do not define the injectate or dose. All those who received an injection matriculated to surgery. None of the 129 patients who received a LESI obtained “substantial relief” post-injection.
Yang 2015, Retrospective	1-2 level lumbar decompression; 63,005, 63,030, 63,047	Medicare PearlDiver Database; 2005-12	125,476	Medicare patients over age 65 who had a lumbar epidural steroid injection within 1 year of 1-2 level lumbar decompression Excluded: Multi-level decompressions (except 63,005), revision surgeries	64,483, 62,311	SSI within 90 days of index surgery; ICD-9: 998.5, 998.51, 998.59; CPT 20,005, 22,015	Database study with inherent accuracy concerns. Unable to rule out bias in patient selection or surgical indication. Unknown data on the number of previous LESI's, steroid dose, level of injections, type of steroid, additional steroid usage, surgical complexity.
Zusman 2015, Retrospective	Thoracic and/or lumbar arthrodesis	Single Site, Portland, OR; 2007-10	289	Elective thoracic and/or lumbar arthrodesis who had completed pre-operative and 90 day postoperative outcome testing (SF-12, ODI). Excluded: trauma, tumor, infection.	Patient reported preoperative spinal injection	Surgical wound complications included hematoma, seroma, and infection requiring an unplanned reoperation within 30 days of index surgery	Defining ESI as “patient reported spinal injection” likely induces recall bias which may impact the results. Unclear when the injection was given preoperatively or what injection was specifically given. Study does not separate between thoracic surgical intervention and lumbar surgical intervention
Hartveldt 2016, Retrospective	Single or multilevel lumbar laminectomy with or without arthrodesis; 22,612, 22,558, 22,630, 22,808, 22,810 for arthrodesis; 63,047, 63,030, 22,630, 63,005, 63,017 for laminectomy	Multi Site, Boston, MA; 2005-15	5,311	18 y/o with at least 90 days of clinical follow up. Excluded: tumor, fracture, trauma, pseudoarthrosis, pre-existing infection	62,311, 0217T, 0230T, 0231T, 64,483, and 64,484	Postoperative SSI as symptoms clinically consistent requiring an incision and drainage intervention performed in the operating room	Underpowered with 5,311 participants. “To detect this difference…we would have needed a sample size of 30,214 patients.” 0217T is a CPT code for facet injections, not LESI. Therefore, the study's results may be confounded by the inclusion of procedures that do not appear to have been intended to be included.
Cancienne 2017, Retrospective	ACDF (22,554, 22,551, 22,585, 63,076, ICD-9 81.02) PCF (22,590, 22,600, ICD-9 81.03)	Medicare PearlDiver Database; 2005-12	Total: 317,733 ACDF: 254,863 PCF: 62,870	Patients w/ PCF or ACDF divided by time from CESI and matched with controls in the same timing sub-cohorts Excluded: Fusions above C2 (22,590, 22,595), revision surgeries	64,479, 62,310	Post op infection within 90 days CPT: 20,005, 10180, 21,501 ICD-9: 998.5, 998.51, 998.59, 996.67, 996.69	Database study with inherent accuracy concerns. Unable to rule out bias in patient selection or surgical indication. Unknown data on the number of previous CESI's, steroid dose, level of injections, type of steroid, additional steroid usage, surgical complexity, instrumentation specifics.
Seavey 2017, Retrospective	1-2 Level lumbar laminectomy/decompression: 63,005, 63,030, 63,047, 63,056	Military Health System Data Repository; 2009-14	6,535	Pts w/ Lumbar ESI prior to single-level lumbar decompression Excluded: Multi-level decompressions (with the exception of 63,005), revision surgeries, prior infection, tumor; those who had prior facet injections	0230T, 64,483, 64,484	SSI within 90 days post op ICD-9 codes :998.51, 998.59	Database study with inherent accuracy concerns. Unable to rule out bias in patient selection or surgical indication. Unknown data on the number of previous LESI's, steroid dose, level of injections, type of steroid, additional steroid usage, surgical complexity.
Singla 2017, Retrospective	1-2 level posteior lumbar spinal fusion; 22,612, 22,614, 22,633, 22,630	Medicare PearlDiver Database; 2005-12	88,540	Medicare patients over age 65 who had a lumbar epidural steroid injection within 1 year of 1-2 level lumbar fusion Excluded: revision surgeries	64,483, 62,311	SSI within 90 days of index surgery; ICD-9: 998.5, 998.51, 998.59, 996.67; CPT 20,005, 22,015	Database study with inherent accuracy concerns. Unable to rule out bias in patient selection or surgical indication. Unknown data on the number of previous LESI's, steroid dose, level of injections, type of steroid, additional steroid usage, surgical complexity, instrumentation specifics.
Ozturk 2018, Retrospective	Microdiscectomy	Single Site, Turkey; 2011-15	66	Pts who had undergone unilateral, single-level lumbar microdiscectomy due to extruded or sequestrated lumbar discs Excluded: BMI >30, Diabetes, Renal Failure, ischemic heart or cerebrovascular disease	Transforaminal anterior epidural steroid injection (TAESI); 80 mg triamcinolone + 3 mL .5% bupivacaine	No infection definition	No infections reported in epidural or control groups Only study which defines ESI contents
Donnally 2018, Retrospective	1 level decompression; 63,030, 63,047	Medicare PearlDiver Database; 2005-14	16,180	Medicare patients over age 65 who had a lumbar epidural steroid injection within 1 year of 1 level lumbar decompression Excluded: fusion, revision surgeries	64,483, 62,311	SSI within 90 days of index surgery; ICD-9: 998.5, 998.51, 998.59; 996.67; CPT 20,005, 22,015	Database study with inherent accuracy concerns. Unable to rule out bias in patient selection or surgical indication. Unknown data on the number of previous LESI's, steroid dose, level of injections, type of steroid, additional steroid usage, surgical complexity.
Pisano 2019, Retrospective	Lumbar arthrodesis 0195T, 22,533, 22,558, 22,612, 22,630, 22,633	Military Health System Data Repository; 2009-14	3,139	Patients who have undergone lumbar spine surgery with and without lumbar corticosteroid injection before (facet and epidural injections included)	Lumbar ESI- 0230T, 64,483, 64,484; Lumbar Facet Injection- 0216T, 0217T, 64,475, 64,476, 64,493	ICD-9 codes 998.51 and 998.59	Despite including facet interventions, published raw data allows for that data to be excluded. Database study with inherent accuracy concerns. Unable to rule out bias in patient selection or surgical indication. Unknown data on the number of previous LESI's, steroid dose, level of injections, type of steroid, additional steroid usage, surgical complexity.
Koltsov 2020, Retrospective	Lumbar decompression, fusion, discectomy. CPT/ICD codes not published	IBM MarketScan Database; 2007-15	220,020	Patients with disc herniation or stenosis, or both who underwent lumbar decompression, fusion or discectomy. Excluded: reoperations, neoplasms, intraspinal abscesses, osteomyelitis, discitis, fracture, dislocation, vehicular accidents, inflammatory spondyloarthropathies, rheumatoid arthritis	64,483, 64,484, 62,311; 0-30 days, 31-60, 61-90, 91-365 days	Post op infection within 90 days from index using “codes published previously.”	They did not separate results by fusion or non-fusion surgical interventions. Therefore, the data was not applicable for Meta-Analyses sub-cohorts which looked exclusively at fusion or non-fusion. Database study with inherent accuracy concerns. Unable to rule out bias in patient selection or surgical indication. Unknown data on the number of previous LESI's, steroid dose, level of injections, type of steroid, additional steroid usage, surgical complexity, instrumentation specifics.
Li 2020, Prospective	Posterior lumbar multi-level fusion for degenerative disc disease	Single Site, China; 2015-19	2,557	Diagnosis of lumbar disc herniation or another degenerative lumbar spine disease con- firmed by radiological examination in the setting of chronic low back pain associated with radicular symptoms >6 months; and participation in medical therapy or physical rehabilitation for >3 months without improvement Excluded: h/o minimally invasive procedures not performed in the OR, lumbar surgery, spinal infection, those who did not follow up	Lumbar transforaminal epidural injection of lidocaine with and without steroid	Surgical site infection related to the operation.	3+ levels of fusion on avg Unclear injection selection method. Moreover, all patients who were injected eventually had surgery. Some injections had steroids, other did not; it is not clear on how this decision was made
Kreitz 2020, Retrospective	All elective lumbar fusion or decompression for radiculopathy and/or spinal stenosis; 63,030, 63,047, 22,612 with minimum 90 day follow up	Single Site, Philadelphia, PA; 2000-17	Total: 15,001 Decompression: 9,903 Fusion: 5,108	All elective lumbar spine procedures performed for a diagnosis of lumbar radiculopathy and/or spinal stenosis with minimum 90 days follow up. Excluded: trauma, pre-existing infection, tumor, and revisions	62,311, 64,475, 64,483, 64,493	Post-operative SSI requiring reoperation <90 days; 996.67, 998.12, 998.31, 998.32, 998.59, T81.31XA, T81.32XA, T84.7XXA	64,475 and 64,493 are CPT codes for facet injections, not ESI. Therefore, the results of the study may be confounded by the inclusion of procedures which do not appear to have been intended to be included.
Wadhwa 2021, Retrospective	CPT for 1-2 level cervical spine surgery: 22,551 22,552 22,554 22,585 22,600 22,614 63,001 63,015 63,020 63,035 63,040 63,043 63,045 63,048 63,050 6,305 63,075 63,076 63,081 63,082	IBM MarketScan Database; 2007-16	59,926	Patients who had cervical degenerative disease and cervical surgery; ICD 9 for cervical degenerative disease 722.0, 722.4, 723.0-723.5, 721.0, 721.1, 722.71, 722.91, and cervical myelopathy 721.1, 722.71 Exclude: <18 y/o, >2 level procedures, tumor or trauma	62,310, 62,311, 64,479, 64,480, 64,483, 64,484, 64,470, 64,472, 64,475, 64,476, 62,318, 62,319	Surgical site infection is based on re-operation within 90 days and ICD coding which was not listed in the published supplement	Injections included, but did not stratify for, cervical ESI, lumbar ESI, epidural catheter placement, and facet interventions. Database study with inherent accuracy concerns. Unable to rule out bias in patient selection or surgical indication. Unknown data on the number of previous LESI's, steroid dose, level of injections, type of steroid, additional steroid usage, surgical complexity, instrumentation type.
Shakya 2022, Retrospective	Lumbar discectomy	Single Center, India; 2017-20	315	21-65 y/o + single-level disc herniation of lumbar spine + at least follow up beyond 6 months + operated via minimally invasive approach Excluded: trauma, tumor, infection, revision, fusion, concomitant cervical spine pathology, spondyloarthropathies, ILESI or caudal	TFESI only	Does not define surgical site infection, but reports that infection was obtained by review of hospital records	They do not define the injectate or dose. All those who received an injection matriculated to surgery. None of the 129 patients who received a LESI obtained “substantial relief” post injection.

Re-arranged by year

We state this because there is a surgical sparing benefit from ESI depending on the pathology, chronicity of symptoms, and the specific injection/injectate used. Some studies demonstrate that surgical sparing benefits may be as high as 80% [[30], [31], [32], [33], [34]]. The number needed to treat (NNT) for an epidural steroid injection in the appropriate clinical setting is, at worst 3 [32,35]. The number needed to harm (NNH), meaning the number of patients who undergo an epidural steroid injection then develop an SSI which per our study may be attributed to that epidural steroid injection is 111 patients. When balancing the surgical sparing benefit of an ESI to the OR identified in our research of postoperative SSI, readers should feel strengthened in our recommendation to consider ESI before moving forward with surgical intervention in the appropriately selected patients.

Interestingly, upon reviewing our selected articles with the knowledge of the potential surgical sparing benefit of ESI relative to the risk of postoperative SSI, it was discovered that somewhere between 9% and 46% of patients trialed an ESI before moving forward with surgical intervention in the studies which made such data available [7,8,10,[14], [15], [16], [17], [18], [19],^30^]. These percentages felt low relative to what we expected, especially given that the patients tended to skew towards single-level disc herniations or stenotic lesions. We would opine that using even the most conservative estimates of the surgical sparing benefit of an ESI, the percentage of patients trialing an ESI should be higher.

There has yet to be a published prospective study from the United States on this topic. Despite this, preoperative ESI use is ubiquitous. To inform better practice and policy regulations involving the intersection of ESI and spinal surgery, researchers ask a more specific question and follow the results prospectively. If designed appropriately, such a study can alter practice habits.

## Conclusion

Our analysis shows a small, albeit statistically significant, association between preoperative ESI and postoperative lumbar SSI may exist. The relationship may be time-dependent as the statistical strength of the association decreased with time from injection to surgery. However, the odds ratios produced, while statistically significant, are close enough to 1.0 regardless of stratification by surgery type, database used, or specific injection that clinically the effect size is “small” or “weak.” At worst, the NNT for an ESI in the appropriate clinical setting is 3. The number need to harm (NNH), meaning the number of patients who undergo an ESI and then develop a SSI, which per our study, may be attributed to that ESI, is 111 patients. Ultimately, the surgical sparing potential from an ESI outweighs whatever SSI risk exists based on our findings.

## Declaration of competing interest

The authors declare no financial or professional conflicts of interests with regards to the contents of this manuscript.

## Appendix Supplementary materials

Image, application 1

## References

[1] Koltsov JCB, Smuck MW, Alamin TF, Wood KB, Cheng I, Hu SS. (2021). Preoperative epidural steroid injections are not associated with increased rates of infection and dural tear in lumbar spine surgery. Eur Spine J Off Publ Eur Spine Soc Eur Spinal Deform Soc Eur Sect Cerv Spine Res Soc.

[2] Zhou J, Wang R, Huo X, Xiong W, Kang L, Xue Y. (2020). Incidence of surgical site infection after spine surgery: a systematic review and meta-analysis. Spine.

[3] Schweizer ML, Cullen JJ, Perencevich EN, Vaughan Sarrazin MS (2014). Costs associated with surgical site infections in veterans affairs hospitals. JAMA Surg.

[4] Yeramaneni S, Robinson C, Hostin R. (2016). Impact of spine surgery complications on costs associated with management of adult spinal deformity. Curr Rev Musculoskelet Med.

[5] Kazarian GS, Steinhaus ME, Kim HJ. (2022). The Impact of corticosteroid injection timing on infection rates following spine surgery: a systematic review and meta-analysis. Glob Spine J.

[6] Patel HA, Cheppalli NS, Bhandarkar AW, Patel V, Singla A. (2022). Lumbar spinal steroid injections and infection risk after spinal surgery: a systematic review and meta-analysis. Asian Spine J.

[7] Zusman N, Munch JL, Ching A, Hart R, Yoo J. (2015). Preoperative epidural spinal injections increase the risk of surgical wound complications but do not affect overall complication risk or patient-perceived outcomes. J Neurosurg Spine.

[8] Hartveldt S, Janssen SJ, Wood KB (2016). Is there an association of epidural corticosteroid injection with postoperative surgical site infection after surgery for lumbar degenerative spine disease?. Spine.

[9] Yang S, Werner BC, Cancienne JM (2016). Preoperative epidural injections are associated with increased risk of infection after single-level lumbar decompression. Spine J Off J North Am Spine Soc.

[10] Seavey JG, Balazs GC, Steelman T, Helgeson M, Gwinn DE, Wagner SC. (2017). The effect of preoperative lumbar epidural corticosteroid injection on postoperative infection rate in patients undergoing single-level lumbar decompression. Spine J Off J North Am Spine Soc.

[11] Singla A, Yang S, Werner BC (2017). The impact of preoperative epidural injections on postoperative infection in lumbar fusion surgery. J Neurosurg Spine.

[12] Donnally CJ, Rush AJ, Rivera S (2018). An epidural steroid injection in the 6 months preceding a lumbar decompression without fusion predisposes patients to post-operative infections. J Spine Surg Hong Kong.

[13] Ozturk S, Akgun B, Erol FS, Onal SA, Kaplan M. (2018). Intraoperative results and postoperative clinical outcomes of lumbar microdiscectomy in patients who previously received a transforaminal anterior epidural steroid injection for lumbar radiculopathy. Turk Neurosurg.

[14] Pisano AJ, Seavey JG, Steelman TJ, Fredericks DR, Helgeson MD, Wagner SC. (2020). The effect of lumbar corticosteroid injections on postoperative infection in lumbar arthrodesis surgery. J Clin Neurosci Off J Neurosurg Soc Australas.

[15] Kreitz TM, Mangan J, Schroeder GD (2021). Do Preoperative epidural steroid injections increase the risk of infection after lumbar spine surgery?. Spine.

[16] Wadhwa H, Varshneya K, Stienen MN, Veeravagu A. (2021). Do epidural steroid injections affect outcomes and costs in cervical degenerative disease? A Retrospective MarketScan Database Analysis. Glob Spine J.

[17] Shakya A, Sharma A, Singh V (2022). Preoperative lumbar epidural steroid injection increases the risk of a dural tear during minimally invasive lumbar discectomy. Int J Spine Surg.

[18] Li P, Hou X, Gao L, Zheng X. (2020). Infection risk of lumbar epidural injection in the operating theatre prior to lumbar fusion surgery. J Pain Res.

[19] Cancienne JM, Werner BC, Puvanesarajah V (2017). Does the timing of preoperative epidural steroid injection affect infection risk after ACDF or posterior cervical fusion?. Spine.

[20] Wang T, Wang H, Yang DL, Jiang LQ, Zhang LJ, Ding WY. (2017). Factors predicting surgical site infection after posterior lumbar surgery: A multicenter retrospective study. Medicine (Baltimore).

[21] Farshad M, Burgstaller JM, Held U, Steurer J, Dennler C. (2018). Do preoperative corticosteroid injections increase the risk for infections or wound healing problems after spine surgery?: a Swiss Prospective Multicenter Cohort Study. Spine.

[22] Kauffman CP. (2016). Return to the operating room following decompression surgery-infection or not infection? That is the question. Spine J Off J North Am Spine Soc.

[23] Chen H, Cohen P, Chen S. (2010). How big is a big odds ratio? Interpreting the magnitudes of odds ratios in epidemiological studies. Commun Stat - Simul Comput.

[24] Rosenthal JA. (1996). Qualitative descriptors of strength of association and effect size. J Soc Serv Res.

[25] Bolognesi MP, Habermann EB. (2022). Commercial claims data sources: PearlDiver and Individual Payer Databases. JBJS.

[26] About PearlDiver Services. PearlDiver. Accessed November 19, 2022. https://pearldiverinc.com/about-us/.

[27] Madenci AL, Madsen CK, Kwon NK (2019). Comparison of Military Health System Data Repository and American College of Surgeons National Surgical Quality Improvement Program-Pediatric. BMC Pediatr.

[28] Yoshihara H, Yoneoka D. (2014). Understanding the statistics and limitations of large database analyses. Spine.

[29] Brookhart M, Stürmer T, Glynn R, Rassen J, Schneeweiss S. (2010). Confounding control in healthcare database research: challenges and potential approaches. Med Care.

[30] Koltsov JCB, Smuck MW, Zagel A (2019). Lumbar epidural steroid injections for herniation and stenosis: incidence and risk factors of subsequent surgery. Spine J.

[31] Ghahreman A, Ferch R, Bogduk N. (2010). The efficacy of transforaminal injection of steroids for the treatment of lumbar radicular pain. Pain Med.

[32] MacVicar J, King W, Landers MH, Bogduk N. (2013). The effectiveness of lumbar transforaminal injection of steroids: a comprehensive review with systematic analysis of the published data. Pain Med.

[33] Riew KD, Yin Y, Gilula L (2000). The effect of nerve-root injections on the need for operative treatment of lumbar radicular pain. A prospective, randomized, controlled, double-blind study. J Bone Joint Surg Am.

[34] Radcliff K, Hilibrand A, Lurie JD (2012). The impact of epidural steroid injections on the outcomes of patients treated for lumbar disc herniation: a subgroup analysis of the SPORT trial. J Bone Joint Surg Am.

[35] Nandi J, Chowdhery A. (2017). A randomized controlled clinical trial to determine the effectiveness of caudal epidural steroid injection in lumbosacral Sciatica. J Clin Diagn Res JCDR.

