# Supplementary material for: The association between preoperative epidural steroid injections and postoperative cervical and lumbar surgical site infections: A systematic review and meta-analysis
Source: N Am Spine Soc J. 2024 Jun 5;19:100334. doi: 10.1016/j.xnsj.2024.100334 (PMC11339057; doi:10.1016/j.xnsj.2024.100334)
Supplement: Supplementary file 1 [file mmc1.docx]

PubMed

10/24/2022 = 658

(corticosteroid injection OR epidural injection OR steroid injection OR epidural steroid injection) AND (spine surgery OR lumbar spine surgery OR cervical spine surgery OR decompression OR fusion OR arthrodesis) AND (infection OR postoperative complication OR surgical site infection)

Cochrane DSM

10/24/2022 = 91

(corticosteroid injection or epidural injection or steroid injection or epidural steroid injection or corticosteroid injections or epidural injections or steroid injections or epidural steroid injections or steroids) AND (spine surgery or spinal surgery or lumbar spine surgery or cervical spine surgery or decompression or fusion or arthrodesis) AND (adverse effects or side effects or complications or risk or risks or infection or infections)

Scopus

10/25/2022 = 554

( TITLE-ABS-KEY ( "corticosteroid injection" OR "epidural injection" OR "steroid injection" OR "epidural steroid injection" OR "corticosteroid injections" OR "epidural injections" OR "steroid injections" OR "epidural steroid injections" OR ( ( triamcinolone OR dexamethasone OR "methyl-prednisolone" ) AND ( injections OR injection ) ) ) AND TITLE-ABS-KEY ( "spine surgery" OR "spinal surgery" OR "lumbar spine surgery" OR "cervical spine surgery" OR decompression OR fusion OR arthrodesis ) AND TITLE-ABS-KEY ( "adverse effects" OR "adverse effect" OR "side effects" OR "side effect" OR complication OR complications OR risk OR risks OR infection OR infections ) )
